# *miR-351* promotes atherosclerosis in diabetes by inhibiting the ITGB3/PIK3R1/Akt pathway and induces endothelial cell injury and lipid accumulation

**DOI:** 10.1186/s10020-022-00547-9

**Published:** 2022-09-30

**Authors:** Hong Li, Dan Song, Qihui Liu, Linlin Li, Xiaoshi Sun, Jiamei Guo, Dianlian Li, Ping Li

**Affiliations:** 1 Department of Cardiovascular, Shengjing Hospital of China Medical University, Shenyang, China; 2 School of Pharmacy, China Medical University, Shenyang, China; 3 Shenyang Open University, Shenyang, China; 4 Department of Endocrinology, Shengjing Hospital of China Medical University, Shenyang, 110004 Liaoning China

**Keywords:** Atherosclerosis, Diabetes mellitus, *miR-351*, *ITGB3*, Aortic endothelial cells

## Abstract

**Background:**

The miR-351 gene is significantly upregulated in diabetic mice with atherosclerosis. However, the mechanism by which its presence is important for the overall disease has not been elucidated. Therefore, this study will investigate the mechanism of miR-351 in the process of diabetes mellitus with atherosclerosis through miR-351 gene knockout mice.

**Methods:**

In this study, miR-351^−/−^ C57BL/6 mice were first induced to form a type 2 diabetes mellitus model with atherosclerosis by STZ injection and a high-fat diet. Pathological tests (oil red O, HE, and Masson staining) combined with biochemical indices (TC, TG, LDL-C, HDL-C, TNF-α, hs-CRP, NO, SOD, MDA, CAT, and GSH-Px) were performed to evaluate the pathological degree of atherosclerosis in each group. Mouse aortic endothelial cells were treated with oxidized low-density lipoprotein (ox-LDL) and 30 mM glucose to establish a diabetic atherosclerosis cell model. Combined with cell oil red O staining and flow cytometry, the effects of silencing miR-351 on lipid accumulation and cell apoptosis in the diabetic atherosclerosis cell model were determined. Fluorescence in situ hybridization was used to detect the localization and transcription levels of miR-351 in cells. The target genes of miR-351 were predicted by bioinformatics and verified by dual-luciferase activity reporting. Western blotting was used to detect the expression levels of phosphorylated inosine 3-kinase regulatory subunit 1 (PIK3R1)/serine/threonine kinase 1 (Akt) and apoptosis-related proteins after transfection with integrin subunit β3 (ITGB3) small interfering ribonucleic acid (siRNA).

**Results:**

The expression of the *miR-351* gene was significantly increased in the high-fat wild-type (HWT) group, and its expression was significantly decreased in the knockout mice. Silencing *miR-351* effectively alleviated atherosclerosis in mice. The levels of *miR-351* expression, apoptosis, lipid accumulation, and oxidative stress in ox-LDL + high glucose-induced endothelial cells were significantly increased. These phenomena were effectively inhibited in lentivirus-infected miR-351-silenced cell lines. Bioinformatics predicted that miR-351-5p could directly target the *ITGB3* gene. Transfection of *ITGB3* siRNA reversed the downregulation of apoptosis, decreased oil accumulation, and decreased oxidative stress levels induced by *miR-351* silencing. In addition, it inhibited the activation of the PIK3R1/Akt pathway.

**Conclusion:**

Silencing miR-351 upregulates ITGB3 and activates the PIK3R1/Akt pathway, thereby exerting anti-apoptosis and protective effects on endothelial cells.

**Supplementary Information:**

The online version contains supplementary material available at 10.1186/s10020-022-00547-9.

## Background

The prevalence of type 2 diabetes mellitus (T2DM) is on the rise worldwide, and its complications are essential factors leading to death (Danaei et al. 2011). It is well known that T2DM promotes atherosclerosis, a process that can contribute to cardiovascular disease. Atherosclerosis, a blood vessel disease caused by hyperlipidemia, accounts for more than half of all deaths in elderly individuals. Cardiovascular complications are the leading cause of diabetes-related morbidity and mortality (Engelen et al. 2017). The leading cause of death and impaired quality of life in patients with type 2 diabetes mellitus (T2DM) is atherosclerotic cardiovascular disease (CVD), the incidence of which is 2 to 4 times higher in these patients than in the general population. An increased risk of cardiovascular disease in patients with diabetes is associated with widespread atherosclerosis. The clinical relationship between diabetes and atherosclerosis is well established. Most clinical and experimental data on the onset of diabetic complications are related to atherosclerosis, suggesting that the effects of chronic hyperglycemia may lead to impaired vascular homeostasis, mainly due to endothelial dysfunction. In addition, diabetes can induce accelerated atherosclerotic lesions to trigger cardiovascular events (Sala et al. 2019). Therefore, clarifying the pathogenesis of T2DM atherosclerosis is of great significance for the treatment of T2DM atherosclerosis and the prevention of cardiovascular diseases.

In recent years, epigenetics has come to be understood as an important form of biological regulation of gene expression and may be the main cause of diabetes and cardiovascular diseases, which are closely related to environmental and lifestyle changes. MicroRNAs (miRNAs) are a class of small noncoding RNAs (22 nt) that regulate multiple cellular processes during life. miRNAs usually function by binding to target genes, resulting in mRNA degradation or translation termination and thus negatively regulating the expression of target genes. There is increasing evidence that miRNAs are involved in the pathogenesis of cardiovascular diseases, including atherosclerosis. miR-126-5p (Schober et al. 2014), miR-92a (Loyer et al. 2014) and miR-155 (Wei et al. 2015) have been reported to prevent or aggravate the development of atherosclerosis (Donaldson et al. 2018). *miR-351* is a newly discovered miRNA that is abnormally expressed in a variety of pathological tissues or cells. It has been reported that *miR-351* is significantly upregulated in serum and endothelial cells of atherosclerotic mice.

Further studies have shown that *miR-351* inhibits endothelial cell proliferation by targeting *STAT3* and subsequently plays an antiangiogenic role. Moreover, transcriptome analysis of diabetic mice revealed that *miR-351* was specifically upregulated in diabetic mice with high lipid induction (Li et al. 2019). These conclusions suggest that *miR-351* may play a role in promoting disease in both diabetic and atherosclerotic mice.

The ITGB3 protein product is the integrin β chain β3. Integrins are complete cell-surface proteins composed of α and β chains. Studies have shown that ITGB3 deficiency can promote atherosclerosis and pulmonary inflammation in high-fat-fed and hyperlipidemic mice (Weng et al. 2003). In *ITGB3* mutant-deficient mice, the number of smooth muscle progenitor cells increased, along with the proliferation and migration of smooth muscle cells. These are significant factors that exacerbate atherosclerosis (Misra et al. 2018). However, ITGB3 has rarely been reported upon in diabetes research. We speculate that it may play a role in atherosclerosis in diabetic patients. ITGB3 is upstream of the PIK3R1/Akt/mTOR signaling pathway in various cell types and is activated when *ITGB3* is overexpressed (Wan et al. 2019). In the atherosclerotic cell model, myricetin activates the PIK3R1/Akt/NOS3 signaling pathways. Thus, attenuated endothelial cell apoptosis is induced by oxidized low-density lipoprotein (ox-LDL) (Qin et al. 2015). Klotho improves oxidative stress induced by oxidized low-density lipoprotein (ox-LDL) by regulating PIK3R1/Akt/NOS3 pathways (Yao et al. 2017). Therefore, we hypothesized that miR-351 might inhibit Pik3r1/Akt pathway activation by weakening ITGB3 expression, thereby promoting atherosclerotic lesions in diabetic patients.

Atherosclerosis is characterized by lipoprotein oxidation, inflammation, and the apoptosis of vascular endothelial cells (Libby et al. 2011). Endothelial cell apoptosis is considered to be one of the significant factors in the pathological process of atherosclerosis. Moreover, endothelial cell apoptosis is stimulated by lipid proteins such as oxidized low-density lipoprotein (ox-LDL) and accelerates plaque deposition (Chen et al. 2000). In this study, a diabetic atherosclerosis model was constructed based on *miR-351* gene knockout mice to clarify the role of *miR-351* in the progression of diabetes-induced atherosclerosis. Meanwhile, the downstream target of *miR-351*, the ITGB3/PIK3R1/Akt pathway, was verified in an ox-LDL + hyperglycemia-induced cell model.

## Methods

### Construction of an atherosclerosis model in type 2 diabetes mellitus mice

The present study used normal 8-week-old male C57BL/6 mice and mmu-miR-351^−/−^ male C57BL/6 mice (*mmu-miR-351* gene knockout mice were commissioned from Fudan University, Shanghai). They were divided into four groups: 1. WT group, wild-type C57BL/6, labeled WT. 2. *miR-351*^*−/−*^ group: mmu-miR-351 knockout C57BL/6, labeled *miR-351*^*−/−*^. 3. HWT group: WT mice + high-fat diet. 4. H + *miR-351*^*−/−*^ group: *miR-351*^*−/−*^ group + high-fat diet. WT and *miR-351*^*−/−*^ mice were the control groups, and HWT and H + *miR-351*^*−/−*^ were the model groups. Each group contained 10 mice (each weighing 20 to 25 g). Referring to the Konstantin method used, mice in the HWT and H + *miR-351*^*−/−*^ groups were used to construct a type 2 diabetes model (Belosludtsev et al. xxxx). The high-fat diet consisted of 20% lard + 2% cholesterol + 80% base feed (Maohua Bio, Shenyang, China). Atherosclerosis is induced by hyperlipidemia, and the whole induction process lasts for 6 months. At the end of the experiment, rats were killed by an overdose of 2% phenobarbital sodium. An eyeball was removed for blood collection, and the aortic arch and abdominal aorta were fixed in neutral formaldehyde solution. Part of the aorta was used for protein and gene detection.

This study was approved by the Shengjing Hospital of China Medical University Animal Care and Use Institutional Committee. The animals were euthanized with an overdose of phenobarbital when they rapidly lost 15–20% of their body weight or when they exhibited signs of eating disorders or organ infections.

### Detection of blood biochemical indices

Peripheral blood was taken for testing between 8:00 am and 10:00 am after 6 months of high-lipid induction (n = 10). Triglycerides (TG, cat no. A110-1-1), low-density lipoprotein cholesterol (LDL-c, cat no. A113-1-1), high-density lipoprotein cholesterol (HDL-c, cat no. A112-1-1), nitric oxide (NO, cat no. A012-1-2), hypersensitivity C protein, hs-CRP (E024-1-1), and serum total cholesterol (TC, cat no. A111-1-1) were detected using kits from the Nanjing Jiancheng Institute of Bioengineering.

### Detection of tissue biochemical indices

Fifty milligrams of aortic arch tissue was weighed, and 450 μL of PBS was added. Then, the tissue was homogenized by a tissue homogenizer to prepare a 5% tissue homogenate. The supernatant was centrifuged, and superoxide dismutase (SOD), malondialdehyde (MDA), catalase (CAT) and glutathione peroxidase (GSH-Px) were detected according to the kit instructions provided by Nanjing Jiancheng Institute of Bioengineering (n = 5).

### Oil red O staining

After the mice were sacrificed, the thoracic aorta was removed and fixed in neutral formaldehyde solution at 4 °C for 24 h. The tissues were then immersed in Oil red O (60% isopropyl alcohol) for 1 h in PBS and stained for 15 min. The remaining oil red O dye was rinsed away with 60% isopropyl alcohol, and the specimens were washed with distilled water 3 times.

### Pathological staining

Aortic arch and abdominal aorta tissue sections were taken from each group. After dewaxing with xylene and soaking in gradient concentrations of alcohol, hematoxylin–eosin staining and Masson staining were performed. Masson staining was performed following the instructions provided for the SolarBio kit (cat no. G1345). The slides were dehydrated and cleared with xylene, covered with a cover plate, sealed with neutral adhesive, and observed under an optical microscope (Leica, DM500). Oil red O staining of cell parts was be performed according to the staining method provided in the manual.

### Quantitative real-time PCR

The gene expression levels of *mmu-miR-351*, endothelin 1 (*EDN1*), prostacyclin (*PTGIS*), angiotensin II (*AGT*), endothelial nitric oxide synthase (*NOS3*), cell adhesion molecule 1 (*ICAM1*), and vascular cell adhesion molecule 1 (*VCAM1*) were detected by real-time PCR. The TRIzol method was used to extract total RNA from mouse tissues or aortic endothelial cells. RNA was reverse-transcribed into cDNA using a reverse transcription kit (gDNA Purge, cat no. E047-01A, Novoprotein, JiangSu, China), and gene expression was detected according to the operating instructions of the NovoStart® SYBR qPCR SuperMix Plus kit (Novoprotein). *Gapdh* was used as an internal reference. For qPCR detection of miRNA, the relative expression level of the *mmu-miR-351-5p* gene was detected by the Guangzhou RiBo Biological miDETECT A Track miRNA qRT‒PCR Starter Kit. The U6 gene was used as the reference gene for miRNA qPCR detection. The relative gene expression levels were calculated by 2^−ΔΔCt^, and 3 replicates were performed for each sample. All the above qPCR tests were performed on an ABI 7500 system. Conventional amplification conditions included predenaturation at 95 °C for 5 min, denaturation at 95 ºC for 20 s; annealing at 60 °C for 20 s for 40 cycles, and extension at 72 °C for 10 min. The primer sequences are shown in Additional file 1: Supplementary materials 1-1.

### Western blot

The following antibodies were used in this study: EDN1 (cat no. 12191-1-AP, Proteintech, USA), PTGIS (cat no. ER64571, HuaBio, Hangzhou, China), AGT (cat no. ET1705-6, HuaBio), NOS3 (cat no. A1548, ABclonal), ICAM1 (cat no. ER1910-98, HuaBio), VCAM1 (cat no. ET1601-18, HuaBio), and GAPDH (cat no. EM1101, HuaBio). Fifty milligrams of aorta or 5 × 10^6^ cells were lysed with 200 μL RIPA protein lysis buffer. Specific methods can be found in the research of Zhang et al. (Zhang et al. 2021). ImageJ was used to quantify each protein band, and GAPDH was used as an internal reference gene for normalization.

For the cell experiment, the required cells were collected. After adding 100 μL of protein lysate, western blotting was performed according to animal experiments. The primary antibodies against ITGB3 (cat no. ET1702-41), Bcl-2 (cat no. ET1702-53), Bax (cat no. EM1203), active Caspase-3 (cat no. ET1602-47), and GAPDH were all purchased from Hua 'an Biotechnology Co., Ltd. Antibodies against PIK3R1 (cat no. 4249S), *p*-PIK3R1 (cat no. 4228S), Akt (cat no. 4691S), and *p*-Akt (cat no. 4060S) were purchased from Cell Signaling Technology. The experimental results were quantitatively analyzed by ImageJ software.

### Construction of the atherosclerotic cell model

Mice were sacrificed by cervical dislocation, and cardiac aorta tissues were isolated from normal C57BL/6 J mice (aged 3–4 weeks). Primary culture of aortic endothelial cells (AECs) was carried out by referring to the method of Pedro et al. (Molina-Sánchez and Andrés 2015). All AEC cells used in the experiment were isolated from the 2^nd^ generation to the 4^th^ generation. Two to four generations of primary AEC were taken and treated with DMEM (containing 10% FBS + 1% penicillin/streptomycin) containing 50 μg/mL oxidized low-density lipoprotein (ox-LDL) and 30 mM glucose (HG) for 48 h to induce the diabetic atherosclerosis model (Wang and Bai 2021; Zhang et al. 2020), denoted as the model group. The Control group was left untreated. The cells were cultured in a constant temperature incubator with 5% CO_2_ at 37 ºC.

### Primary identification of aortic endothelial cells

In cultured primary cells, immunofluorescence detected the expression levels of CD31 and CD34 markers in aortic endothelial cells. First, the primary cells were treated with silver, and after the cells were covered with cover glass, they were fixed with 4% paraformaldehyde. After PBS washing, 1% BSA was added and incubated at room temperature for 30 min. Rabbit anti-mouse CD31 (cat no. ER31219, Hua 'an) and CD34 (cat no. ER0802, Hua 'an) antibodies were added and incubated at 4 ºC overnight. After washing with PBS 3 times, red and green fluorescent-labeled secondary antibodies were added and incubated for 1 h at room temperature. DAPI was used to stain cells for 10 min with 1 μg/mL DAPI. After dropping the anti-fluorescence quenching agent, the tablets were sealed with nail polish. The results were photographed by a fluorescence microscope (Leica, DM500).

### Construction of miR-351-silenced cell lines

The NCBI website (https://www.ncbi.nlm.nih.gov) was searched to obtain the sequence of mmu-miR-351 in mice, and the shRNA sequence was designed through the GPP Web Portal online website (https://portals.broadinstitute.org/gpp/public/), taking the first three higher-score sequences of mmu-miR-351-5p for the carrier construction. Two complementary single-stranded oligonucleotides containing the target sequence were chemically synthesized and annealed. Double-stranded oligonucleotides were inserted between the pLKO-CMV-GFP-puro plasmid *Age*I and *EcoR*I restriction sites, and the linked plasmids were transformed into Escherichia coli DH-5α competent cells for plasmid amplification. Plasmids from positive colonies were verified by RT‒PCR and DNA sequencing.

The recombinant lentivirus was prepared by cotransfection. The lentiviral vectors △8.91 and pVSV-G (10:10:1) were transfected into 293 T cells (X-Tremegene HP DNA transfection reagent, Roche) using the cationic lipid complex method. The cell density was 1 × 10^6^ cells/pore. The cells were divided into a control group (negative control lentiviral vector infection) and a *mmu-miR-351* shRNA 1–3 group (lentiviral vector infection). AECs were seeded into 6-well plates at a density of 5 × 10^5^ cells/well. The next day, cells were infected with viruses at the same titer containing 2 µg/mL polybrene, and after 72 h of infection, the medium was replaced with medium containing 2 μg/mL puromycin. Cell growth was maintained for 7–9 days. The medium was then replaced with medium without puromycin, and some cells from each group were used for qPCR detection of the mmu-miR-351 expression level to determine the silencing efficiency. The primer sequences are shown in Additional file 1: Supplementary materials 1-2.

### Cell transfection

The siRNA sequence of *ITGB3* was designed according to its CDS region and was commissioned to be synthesized by Guangzhou RiboBio. When the convergence degree of lentivirus-infected AECs was 70–90%, the *ITGB3* siRNA (50 nM) was transfected with Lipofectamine RNAiMAX (Thermo Fisher Scientific), and the cells were incubated in a CO_2_ incubator at 37 °C for 48 h. The standard medium was replaced, and a portion of the cells was used for qPCR and western blot detection. The other portion of the cells was treated with 50 μg/mL ox-LDL for 48 h for subsequent experimental detection. The siRNA primer sequences are shown in Additional file 1: Supplementary materials 1-3.

### Measurement of cell viability by MTT

The cells were inoculated into 96-well plates (Gibco-BRL) with 200 μL medium per well and cultured at 37 °C for 24 h. The plates were then treated with medium containing 50 μg/mL ox-LDL and 30 mM glucose for 48 h, and 10 μL MTT (5 mg/mL) was added to each well. The plates were then incubated at 37 °C for 4 h. The medium was removed, 150 μL DMSO (Sigma‒Aldrich) was added to each well, and the plate was shaken for 5 min. A microplate reader (Molecular Devices, USA) was used to perform absorbance measurements at 570 nm.

### DAPI staining

*MiR-351*-silenced and normal AEC cells (1 × 10^5^) were plated in 6-well plates, and 3 cover slides were placed at the bottom of each well plate. When the cells grew to 90% confluence, the medium was replaced with medium containing 50 μg/mL oxidized low-density lipoprotein (ox-LDL) and 30 mM glucose for 48 h. After removing the cover glasses, the cells were fixed with methanol at 4 °C 15 min, washed with PBS 3 times, and then incubated with 1 μg/mL DAPI drops for 10 min. After washing with PBS 3 times, the anti-fluorescence quenching agent was added dropwise, sealed with nail polish, and photographed. The results were obtained with a fluorescence microscope (Leica, DM500).

### Apoptosis detection

Apoptosis was detected by Annexin V-FITC/PI double staining. Lentivirus-infected cells in each group were treated with 50 μg/mL ox-LDL and 30 mM glucose for 48 h by following the instructions of the Solarbio (Beijing, China) Annexin V-FITC/PI apoptosis detection kit (cat no. CA1020). The cell apoptosis rate was detected by an Attune N × T flow cytometer (ABI, USA). The experiment was repeated three times.

### Oil red O staining

A total of 5 × 10^4^ cells from each group were seeded and cultured in 24-well plates. After 24 h, the cells had adhered to the wall, and the convergence degree was approximately 70%. After 48 h, oil red O staining was performed according to the instructions of the oil red O staining kit (cat no. G1262, Solarbio). The cells were imaged with an inverted microscope (Leica).

### Reactive oxygen species (ROS) detection

Superoxide anion formation was detected by dichlorodihydrofluorescein diacetate (DCFH-DA) (Sigma‒Aldrich). The 2nd and 3rd generations of myocardial microvascular endothelial cells were taken and inoculated on cover glasses. After cell adherence, the cells in each group were treated according to their pregroup. After treatment, each group was divided into two EP tubes with no less than 1 × 10^6^ cells in each tube. DCFH-DA solution was added to the cell culture medium at a final concentration of 5 μmol/L and incubated for approximately 30 min. After the cell slides were removed and washed with PBS, one tube was taken from each group, and the ROS content was detected by flow cytometry to determine the degree of oxidative damage to the cells. The fluorescence intensity was analyzed to quantitatively determine the degree of oxidative damage. The excitation wavelength was 504 nm, and the emission wavelength was 529 nm.

### Oxidative stress index detection

Lentivirus-infected cells were treated with high glucose and low-density lipoprotein for 48 h and then digested and collected with trypsin. SOD (A001-3–2), MDA (A003-1–2) and GSH-Px (A005-1–2) indices were detected. All kits were purchased from Nanjing Jiancheng Institute of Biological Engineering.

### *Fluorescence *in situ* hybridization*

Guangzhou RiboBio biosynthesis of the *miR-351-5p* FISH probe was commissioned, FISH hybridization was conducted according to the FISH kit instructions, and the influence of *miRNA-351* expression on AEC cells cultured in slides after treatment with ox-LDL and high-glucose medium was determined.

### Bioinformatics analysis

Genes related to atherosclerosis and vascular disease lipid metabolism disorders were obtained from the GeneCard website. Target gene prediction of the *mmu-miR-351-5p* sequence was performed using miRDB (http://mirdb.org). The obtained disease-related genes were screened by intersection with *mmu-miR-351-5p-*interacting genes to determine the target genes.

### Dual-luciferase reporter assay

Partial sequences of the *ITGB3*-3'UTR containing wild-type and mutated *miR-351* binding sites were cloned into pIS0 luciferase vectors (Promega, Madison, WI, USA) to generate *ITGB3*-WT and *ITGB3*-MUT. pRL-TK was used as an internal reference plasmid. 293 T cells were transfected at 60–80% fusion degree. The *miR-351* mimic and NC mimic (2.5 μL, final concentration of 100 nM) were purchased from Genewiz Company for synthesis. Lipofectamine 2000 was used for transfection. A Promega GloMax was used to measure the dual-luciferase reporting system (Promega). Relative luciferase activity was expressed as the ratio of firefly luciferase activity to Renilla luciferase activity. The primer sequences involved are provided in Additional file 1: Supplementary materials 1-4.

### Statistical methods

In the animal experiments, 3 samples were randomly selected from mice in each group for experimental detection unless otherwise indicated. Cytological tests were performed in triplicate. All data are represented as the mean ± standard deviation. Statistical significance between groups was assessed by a two-tailed unpaired Student’s t test. All results were calculated using GraphPad Prism Software (version Prism 8; GraphPad Software, Inc.). P < 0.05 was considered to indicate a statistically significant difference.

## Results

### Construction of a mouse model of type 2 diabetes mellitus with atherosclerosis

**Fig. 1:**
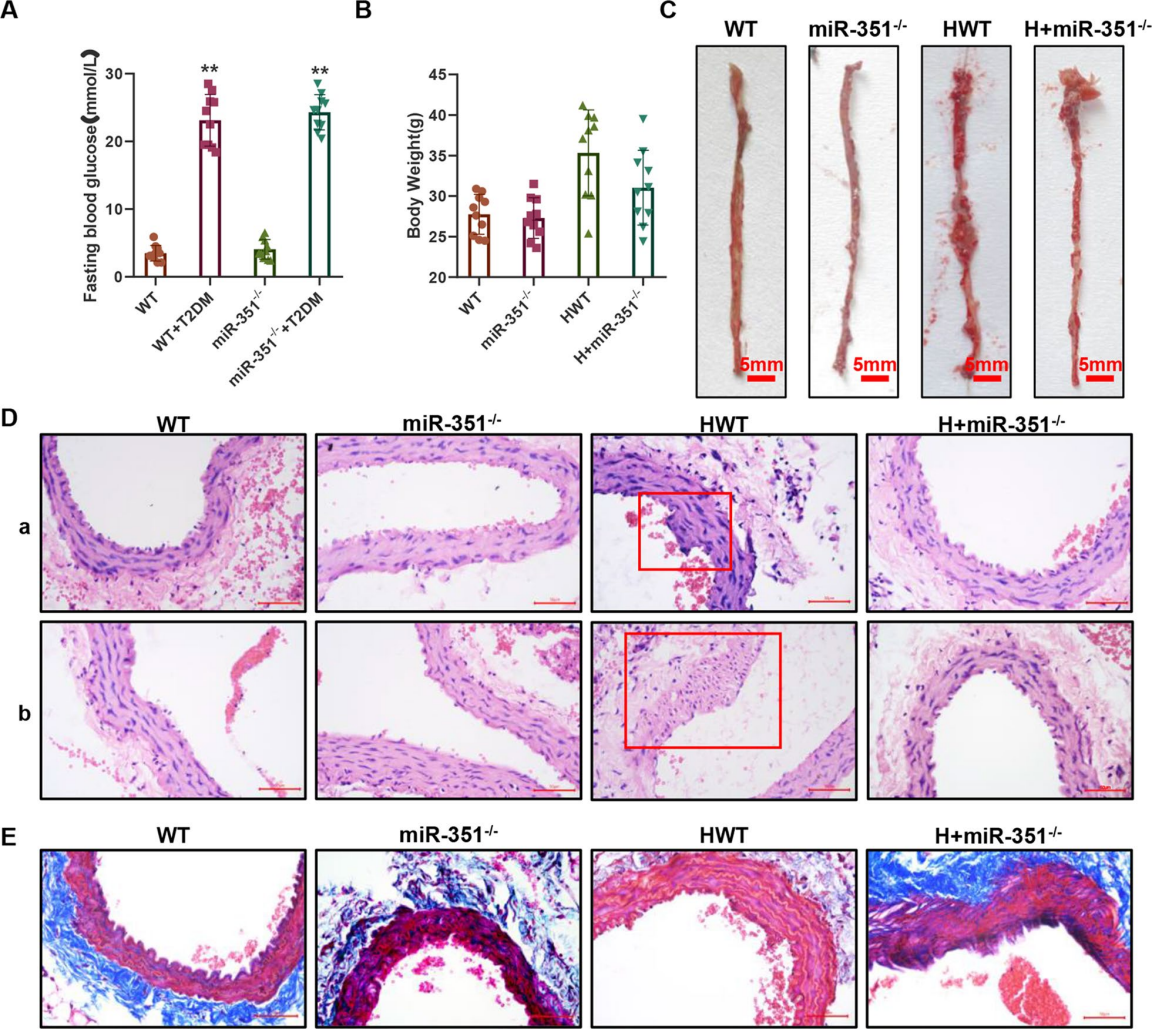
A mouse model of atherosclerosis was established, and pathological examination was conducted. **A** One-touch detection of the fasting blood glucose FBG index in mice. N = 10 mice per group. **B** Weight results of mice in each group. N = 10 mice per group. **C** Oil red O was used to stain the aorta in the thoracic region of mice in each group, and red stain indicated the site of oil presence or plaque formation. **D** HE staining results: The vascular tissue of the abdominal aorta is shown in the upper part, and the aortic arch is shown in the lower part. The scale bar represents 50 μm. **E** Masson trichromatic staining was performed on collagen, with blue representing collagen parts and red representing muscle fibers and other tissues. The scale bar represents 50 μm. Histopathological test, N = 3 mice per group

As shown in Fig. 1A, the fasting blood glucose levels of wild-type and *miR-351* knockout mice were 23.6 ± 3.5 mmol/L and 24.6 ± 2.6 mmol/L, respectively, after treatment with 50 mg/kg STZ for 5 consecutive days, and the FBG indices were both greater than 13.8 mmol/L. The blood glucose values of the WT and *miR-351*^*−/−*^ groups were 3.5 ± 1.2 mmol/L and 4.1 ± 1.5 mmol/L, respectively. At the end of the experiment, the body weight of the HWT group was 32.5 ± 5.4 g, and that of the H + *miR-351*^*−/−*^ group was 29.5 ± 4.5 g (Fig. 1B). Figure 1C shows oil red O staining of the aorta of the thoracic segment of mice in each group. The plaques formed by oil red O staining could hardly be seen in the aortas of the WT and *miR-351*^*−/−*^ groups, while in the HWT group, there were more small red spots of oil patches in the aorta and a few red spots of oil patches in the H + *miR-351*^*−/−*^ group. HE staining of the abdominal aorta (Fig. 1Da) revealed apparent endothelial cell protrusions and abnormal cell proliferation in the HWT group. The H + *miR-351*^*−/−*^, WT, and *miR-351*^*−/−*^ groups showed no significant endothelial bulge or other abnormalities in the abdominal aorta, while the vascular wall was well shaped. HE staining of the mouse aortic arch (Fig. 1Db) showed that vascular damage was not evident in the WT and *miR-351*^*−/−*^ groups. In contrast, vascular endothelial cells at the aortic arch in the HWT group showed evidence of hyperplasia disorder, extensive proliferation of smooth muscle cells, thickening of the wall, and noticeable swelling toward the intima surface. The H + *miR-351*^*−/−*^ group showed thickened vascular walls. Figure 1E shows the Masson staining results; blue indicates collagen, red muscle fiber and other proteins, revealing that the WT and abdominal aorta vascular *miR-351*^*−/−*^ group contains more collagen, while the collagen in the HWT group was obviously reduced. In the surrounding blood vessels around the loss of collagen, the vascular smooth muscle layer was also reduced, and H + *miR-351*^*−/−*^ collagen levels in our HWT group showed apparent improvement.

### Effect of silencing miR-351 on the degree of atherosclerotic injury

**Fig. 2:**
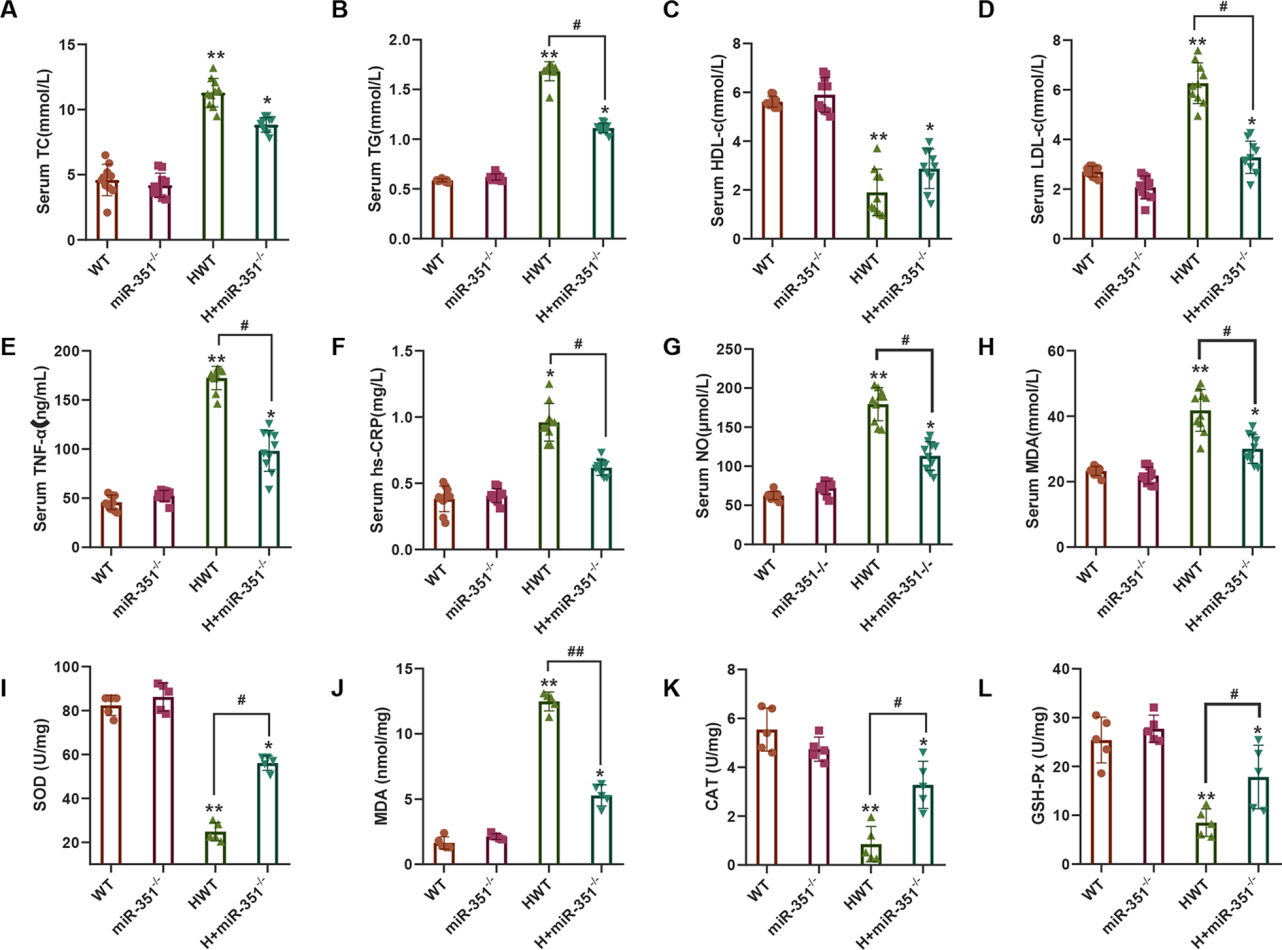
Serum and tissue biochemical tests. **A** Serum total cholesterol (TC) levels were detected. **B** Serum triglyceride (TG) levels were detected. **C** Serum high-density lipoprotein cholesterol (HDL-c) levels were measured. **D** Serum low-density lipoprotein cholesterol (LDL-c) levels were detected. **E** Serum tumor necrosis factor α (TNF-α) was detected. **F** Serum hs-CRP levels were detected. **G** Serum nitric oxide (NO) levels were detected. **H** Serum malondialdehyde (MDA) levels were detected. **I** Detection of superoxide dismutase (SOD) activity in aortic tissue. **J** Detection of MDA levels in aortic tissue. **K** CAT activity in aortic tissue was measured. **L** Glutathione peroxidase (GSH-Px) activity in aortic tissue was detected. **P < 0.01 *P < 0.05, vs. WT group. ^#^P < 0.05 represents the comparison between the two groups of connections. (Serum test, N = 10 mice per group; tissue detection, N = 5 mice per group)

The serum levels of TC (Fig. 2A), TG (Fig. 2B), HDL-c (Fig. 2C), LDL-c (Fig. 2D), TNF-α (Fig. 2E), hs-CRP (Fig. 2F), NO (Fig. 2G), and MDA (Fig. 2H) in each group were detected. The serum levels of TC, TG, LDL-c, TNF-α, hs-CRP, NO, and MDA in the HWT group with atherosclerosis were significantly increased, while the HDL-c levels were decreased markedly and were substantially different from those in the WT and *miR-351*^*−/−*^ groups (P < 0.05). After silencing *miR-351*, the levels of TG, LDL-c, TNF-α, hs-CRP, NO, and MDA in the serum of mice were significantly decreased. In contrast, the level of HDL-c in serum was increased, and the difference was statistically significant compared with that in the HWT group (P < 0.05). The oxidative stress indices SOD (Fig. 2I), MDA (Fig. 2J), CAT, and GSH-Px (Fig. 2K) were measured in the heart tissues of mice in each group. The SOD, CAT, and GSH-Px enzyme activities in the aortic tissue of the HWT group were significantly decreased, and MDA levels were significantly increased compared with those in the WT and *miR-351*^*−/−*^ groups (P < 0.05). *miR-351* silencing of H + in the *miR-351*^*−/−*^ group significantly increased the activities of SOD, CAT, and GSH-Px and decreased MDA production, with significant differences compared with the HWT group (P < 0.05).

### Effects of silencing of miR-351 on the expression of atherosclerotic genes and proteins

**Fig. 3:**
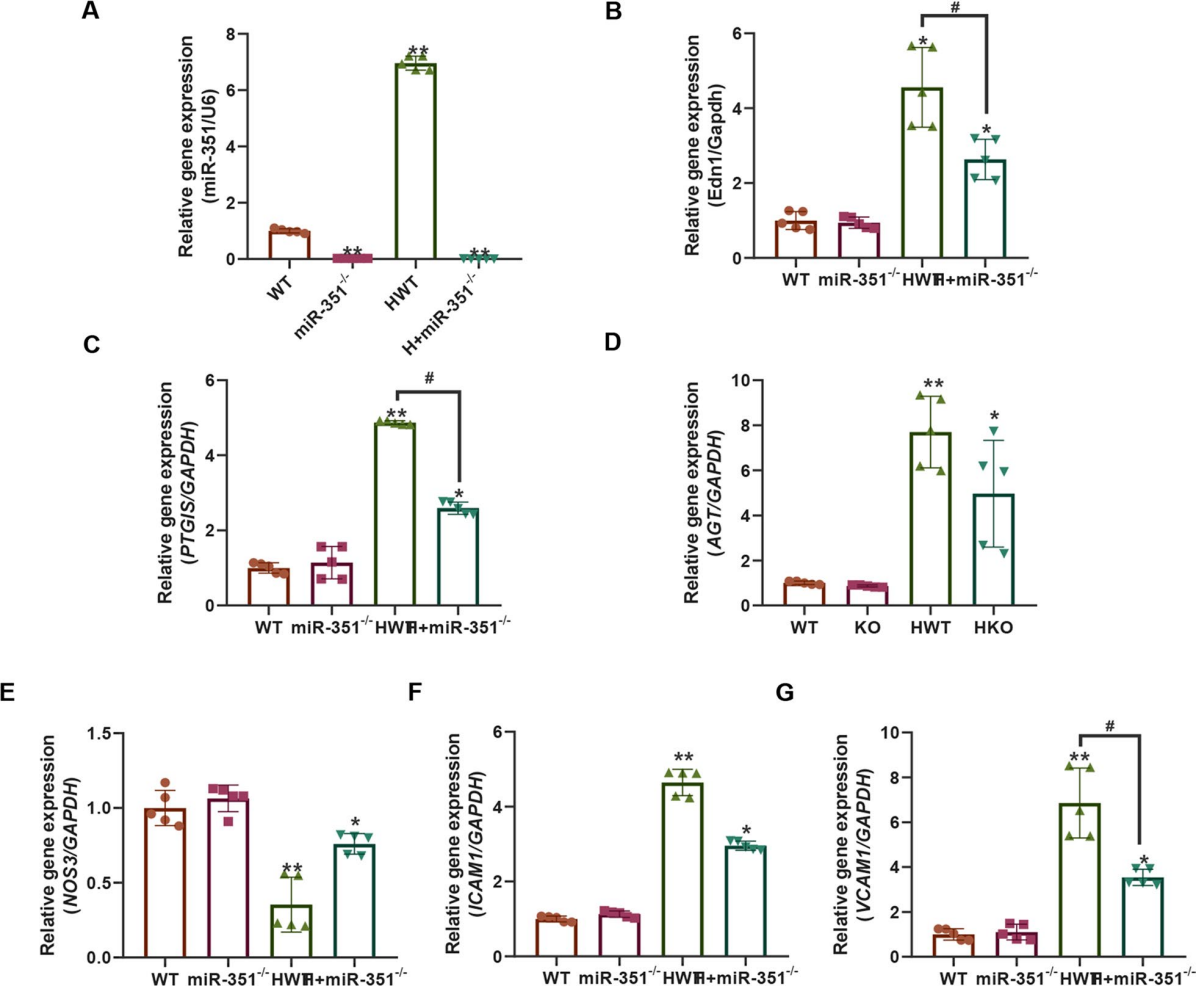
qPCR detection results. **A** *MiR-351* gene expression level in the serum of each group of mice; **B**–**G** Relative expression levels of the *EDN1*, *PTGIS*, *AGT, NOS3*, *ICAM1* and *VCAM1* genes in the aorta of each group. N = 5 mice per group **P < 0.01 *P < 0.05, vs. the WT group. ^#^P < 0.05 represents the comparison between the two groups of connections

**Fig. 4:**
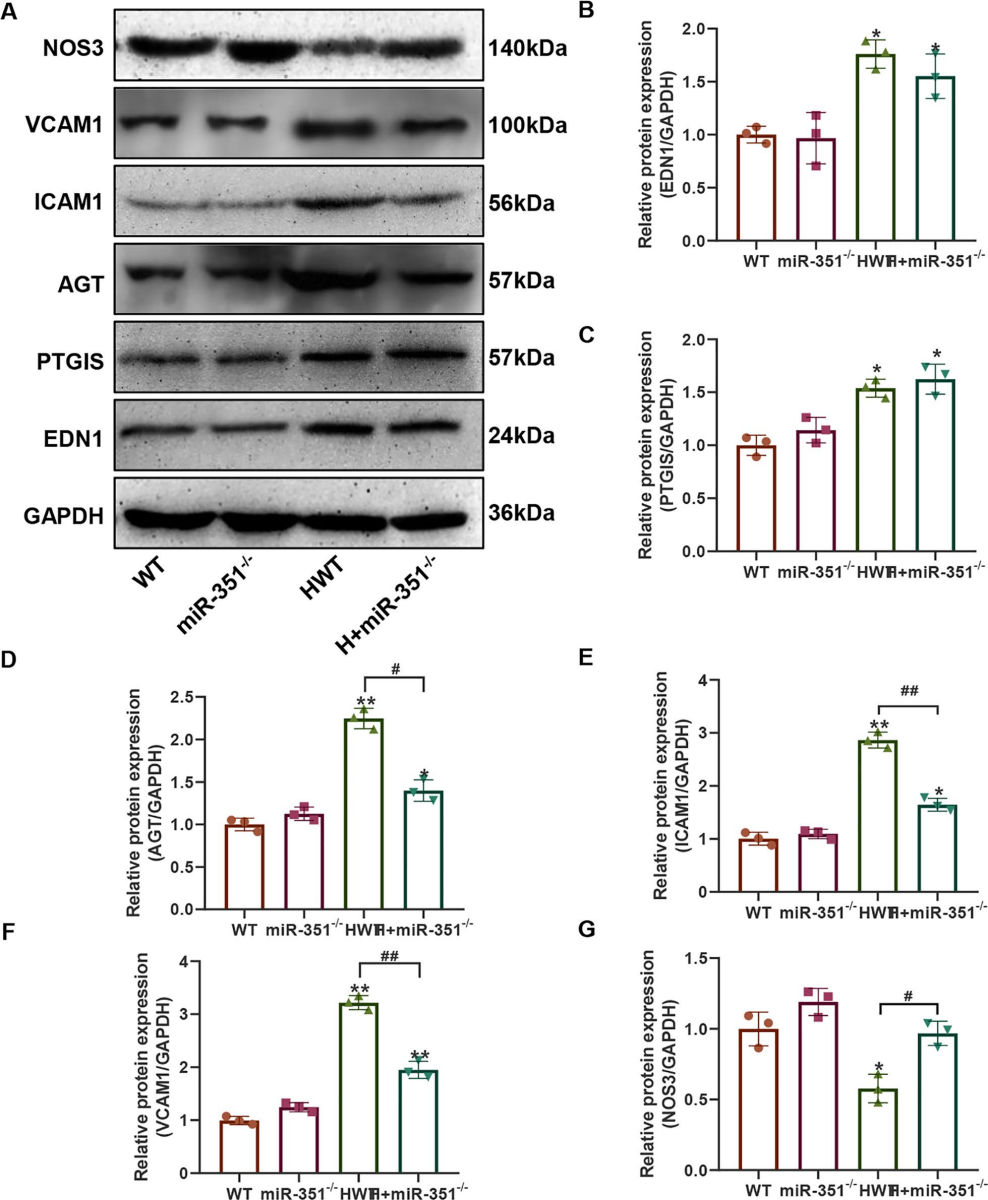
Western blot results of atherosclerotic-related proteins in *miR-351*^*KO*^* mice*.** A** Western blot detection results. **B** ImageJ quantified statistical results of the NOS3 band gray values. **C** ImageJ quantitative statistical results of the VCAM1 densitometry values. **D** ImageJ quantitative statistical results of the ICAM1 band gray values. **E** ImageJ quantitative statistical results of the AGT band gray values. **F** ImageJ quantitative statistical results of the PTGIS band gray values. **G** ImageJ quantitative statistical results of the EDN1 strip gray values. **P < 0.01, *P < 0.05, vs. the WT group. ^#^P < 0.05 represents the comparison between the two groups of connections. N = 3 mice per group

Figure 3A shows the expression level of the *miR-351* gene detected by qPCR, and the results indicated that the expression of the *miR-351* gene was detected in the whole blood of the *miR-351*^*−/−*^ and H + *miR-351*^*−/−*^ groups of mice with *miR-351* gene knockout. Moreover, the *miR-351* gene expression level in the whole blood of the HWT group was higher than that of the WT group (P < 0.01). Subsequently, genes related to the atherosclerosis indicators *EDN1*, *PTGIS*, *AGT*, *NOS3*, *ICAM1*, and *VCAM1* were detected by qPCR (Fig. 3B**–**G). The results showed that compared with the WT and *miR-351*^*−/−*^ groups, the gene expression levels of *EDN1, PTGIS*, *AGT*, *ICAM1*, and *VCAM1* in the HWT group were significantly increased. At the same time, *NOS3* was significantly downregulated, with statistically significant differences (P < 0.01). The H + *miR-351*^*−/−*^ group could effectively recover the expression levels of these genes, making them closer to the gene expression levels of the WT and *miR-351*^*−/−*^ groups, and the *EDN1*, *PTGIS*, and *VCAM1* gene levels in the H + *miR-351*^*−/−*^ group were significantly decreased compared with those in the HWT group, with statistically significant differences (P < 0.05). Then, the expression levels of these proteins were detected by western blotting (Fig. 4A). The quantitative results of the grayscale values of the bands revealed that the protein expression levels of VCAM1, ICAM1, AGT, PTGIS, and EDN1 were all increased (Fig. 4B–G). In contrast, the protein level of NOS3 was significantly decreased. Compared with the WT and *miR-351*^*−/−*^ groups, the difference between groups was statistically significant (P < 0.05). The protein expression levels of EDN1, AGT, ICAM1, and VCAM1 in the H + *miR-351*^*−/−*^ group were significantly lower than those in the HWT group. The difference between the two groups was statistically significant (P < 0.05).

### Construction of the experimental model of atherosclerotic cells

**Fig. 5:**
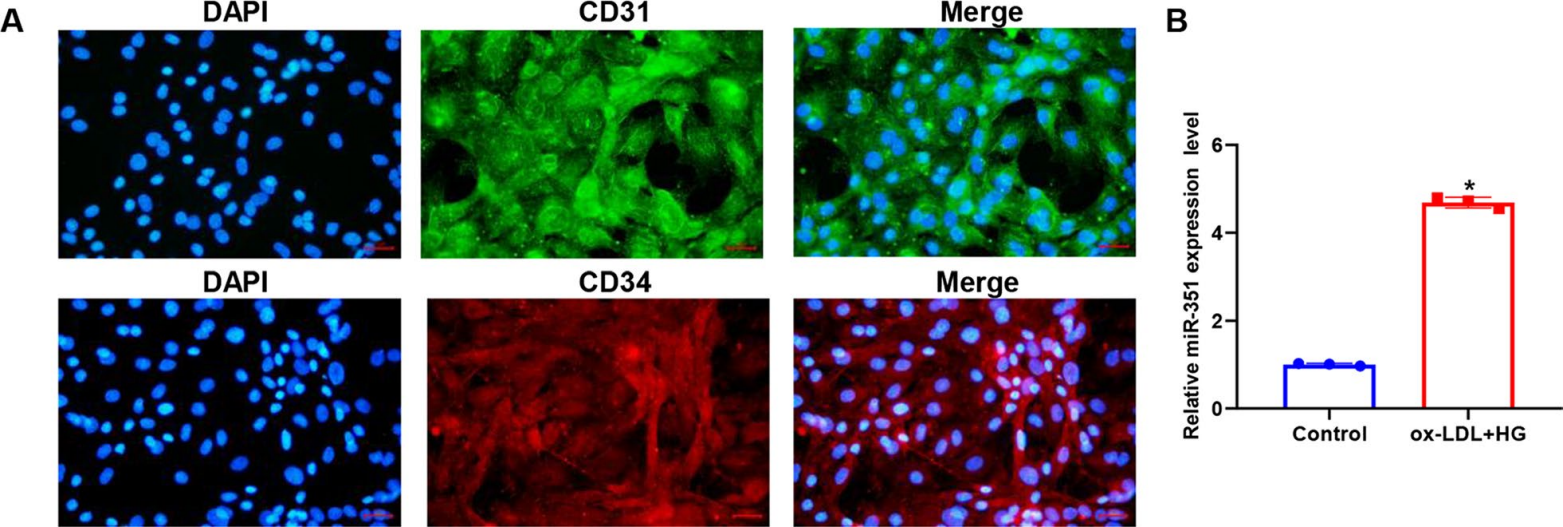
Cell line construction. **A** CD31 and CD34 staining was performed on primary aortic cells of mice by immunofluorescence, and double-positive results indicated that primary culture of main arterial endothelial cells was successful. The scale bar represents 50 μm. **B** qPCR was used to detect the expression levels of ox-LDL and miR-351 in cells induced by 30 mM glucose. *P < 0.01, vs. control group. N = 3 per group

Figure 5A shows the detection of the vascular endothelial cell markers CD31 and CD34 by immunofluorescence in mouse aortic endothelial cells. The CD31 and CD34 proteins were widely expressed in the cultured primary cells. Figure 5B shows the expression level of *miR-351* in cells detected by qPCR after treatment with DMEM containing 50 μg/mL ox-LDL and 30 mM glucose for 48 h. The results showed that *miR-351* was significantly upregulated in atherosclerotic cell models (P < 0.05).

### Effect of miR-351 silencing on cell biological function in the atherosclerotic model

**Fig. 6:**
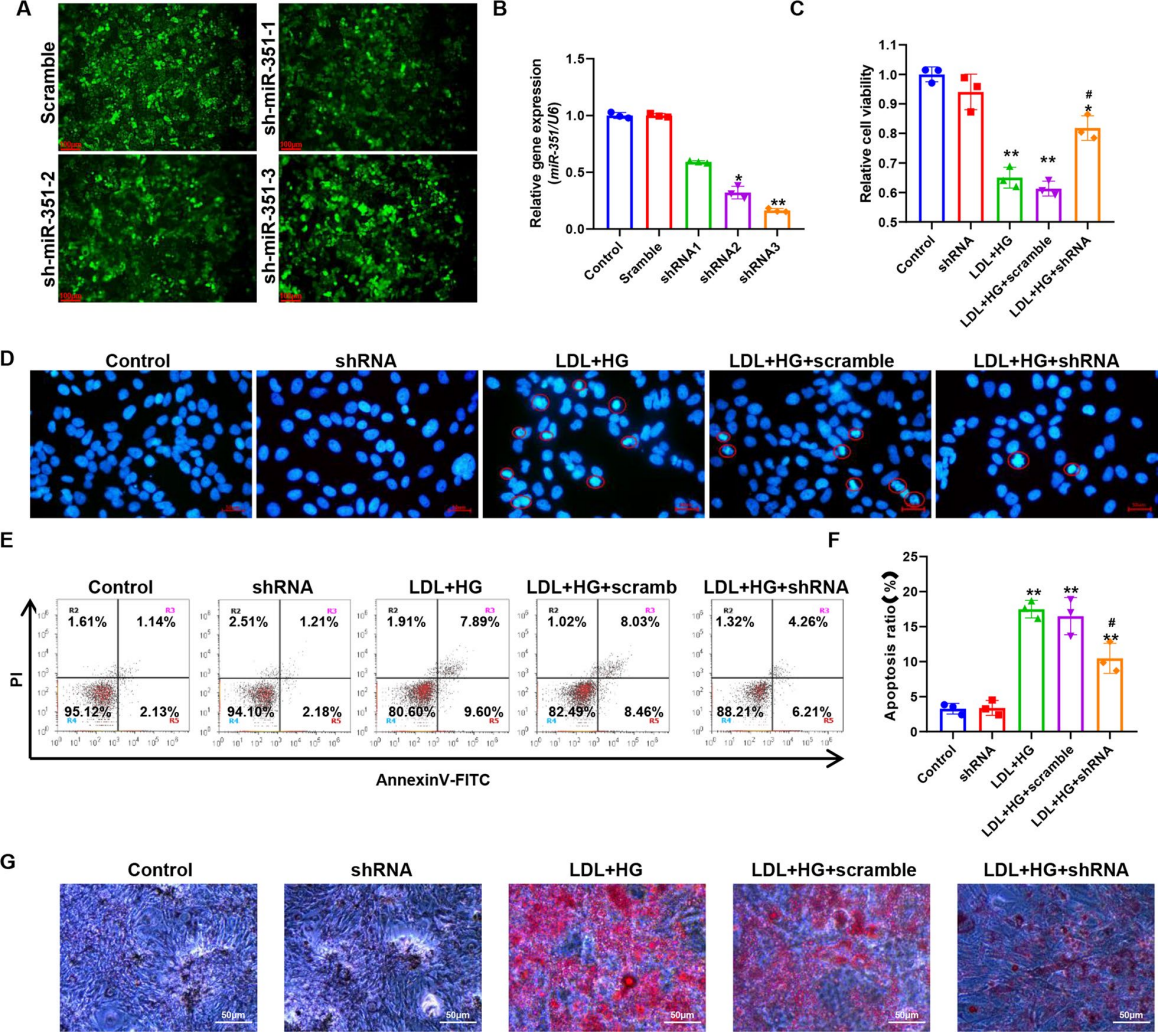
Effect of *miR-351* silencing on the atherosclerotic cell model. **A** Lentivirus was used to infect cells and deliver packaged shRNA plasmids, and the success of infection was determined by fluorescence microscopy. The scale bar represents 100 μm. **B** qPCR was used to detect the silencing efficiency of shRNA on the *miR-351* gene in the three groups. **C** *MiR-351* was treated with ox-LDL + 30 mM glucose for 48 h, and MTT was used to detect the activity of cells in each group. **D** DAPI (1 μg/ml) was used for nuclear staining of cells in each group, and apoptotic cells are shown in red circles. The scale bar represents 50 μm. **E** Flow cytometry was used to detect apoptosis. Annexin V-FITC fluorescence intensity is shown by the horizontal coordinates, and PI staining is shown by the vertical coordinates. Dead cells are present in the R2 quadrant, late apoptotic cells are in the R3 quadrant, normal cells are in the R4 quadrant, and early apoptotic cells are in the R5 quadrant. **F** Flow cytometry was used to detect apoptosis. **G** Oil red O stains cells with oil, and red stain indicates the oil-positive part. The scale bar represents 50 μm. **P < 0.01, *P < 0.05, vs. the control group. ^#^P < 0.05, vs. LDL + HG group. N = 3 per group

**Fig. 7:**
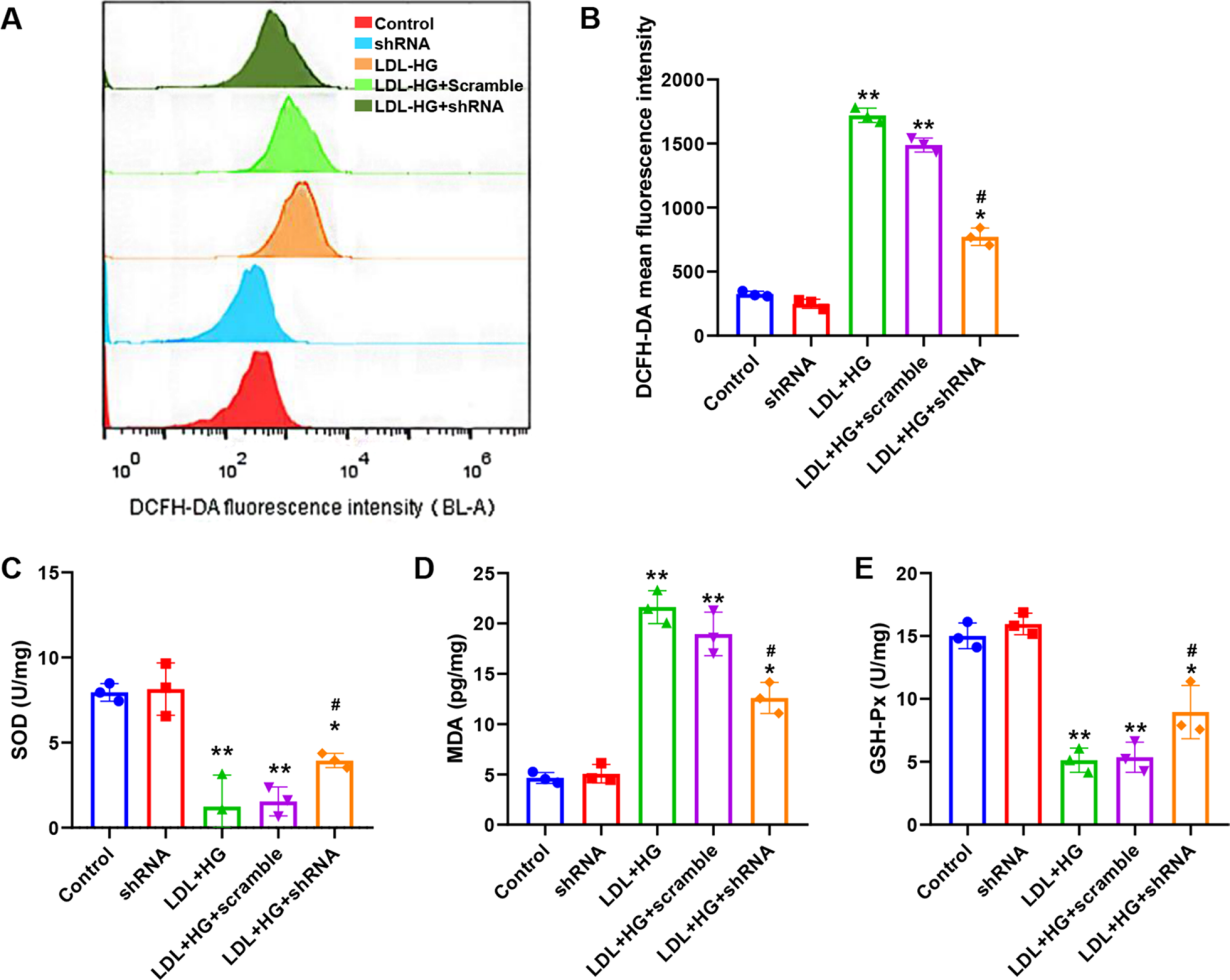
Effect of silencing *miR-351* on the oxidative stress level of cells. **A** The intensity of the DCFH-DA probe was detected by flow cytometry to reflect the ROS level of cells. **B** Quantification of flow cytometry results. **C** Results of SOD level detection in cells. **D** Results of MDA level detection in cells.** E** Cell GSH-Px detection results. **P < 0.01, *P < 0.05, vs. the control group. ^#^P < 0.05, vs. LDL + HG group. N = 3 per group

Figure 6A shows the fluorescence microscopy results after lentivirus infection of primary cells, indicating that the three groups of shRNAs were successfully transferred into cells. Figure 6B shows the silencing efficiencies of the three shRNA pairs against *miR-351* detected by qPCR. The results indicated that shRNA3 had the highest silencing efficiency against *miR-351* (84%). Therefore, shRNA3 was selected for the follow-up experiment and recorded as shRNA. Figure 6C shows the detection of cell activity by MTT. The results indicated that the cell activity of the LDL + HG group decreased significantly after the induction of the atherosclerosis model, which was substantially different from that of the control group (P < 0.01). Compared with the LDL + HG + shRNA group, the *miR-351* silencing group showed significantly upregulated cell activity (P < 0.05), but there was still a significant difference between the control group and the LDL + HG + shRNA group (P < 0.05). Subsequently, DAPI was used to stain the nuclei (Fig. 6D), and we detected many agglutinated nuclei in the LDL + HG and LDL + HG + Scramble groups, characterized by nuclear fragmentation and presenting typical apoptotic bodies. Red circles mark apoptotic cells. In contrast, the amounts of nuclear pyknosis and fragmentation were decreased significantly in the LDL + HG + shRNA group. Figure 6E shows the results of cell apoptosis level detection by flow cytometry. The quantitative results (Fig. 6F) showed that the percentages of cell apoptosis in the LDL + HG and LDL + HG + Scramble groups were 17.49 ± 1.26% and 16.49 ± 2.65%, respectively. There was an extremely significant difference between the control and the LDL + HG groups (P < 0.01). The percentage of apoptotic cells in the LDL + HG + shRNA group was 10.47 ± 2.14%, which was significantly different from that in the control group (P < 0.01), but compared with the LDL + HG group, the percentage of apoptotic cells was significantly reduced, and the difference between the two groups was statistically significant (P < 0.05). Figure 6G shows the results of oil red O staining, which revealed that the oil accumulation degree of miR-351-silenced cells was significantly reduced compared with that of the LDL + HG group. Figure 7A shows the fluorescence intensity of the DCFH-DA probe by flow cytometry. Quantitative results (Fig. 7B) indicated that ROS accumulation increased significantly after ox-LDL + 30 mM glucose treatment compared with the control group, and the difference between the two groups was statistically significant (P < 0.01). Silencing miR-351 can significantly downregulate the fluorescence intensity of DCFH-DA, that is, decrease the accumulation of ROS. Figure 7C–E shows the biochemical detection results of SOD, MDA, and GSH-Px. The activities of the SOD and GSH-Px enzymes were significantly reduced by LDL + HG induction and could restore the activity of antioxidant enzymes to some extent after silencing miR-351 and could reduce the production of MDA.

### Potential targets of miR-351

**Fig. 8:**
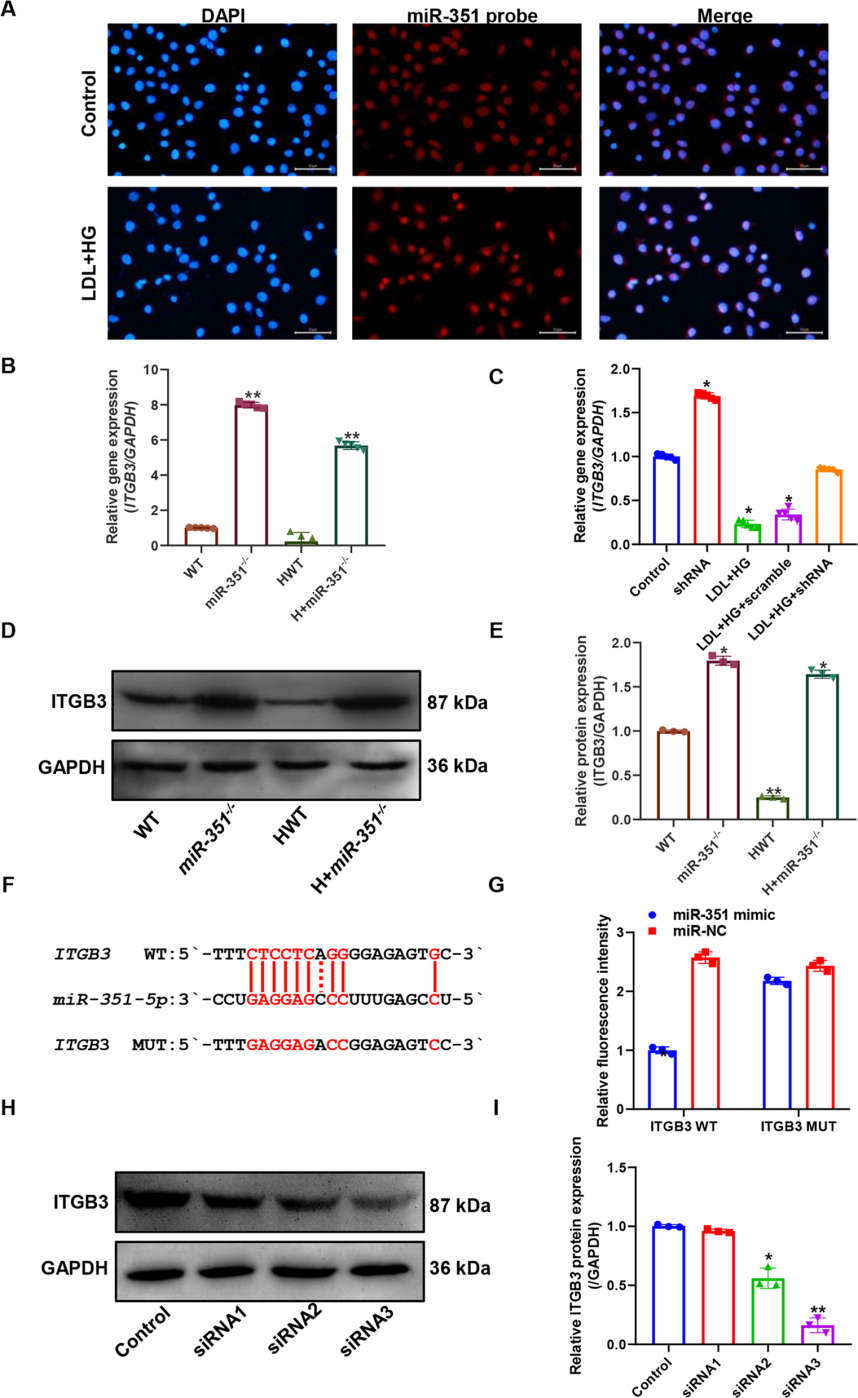
Gene targets of *miR-351*. **A** The localization and expression intensity of *miR-351* in cells were detected by FISH. **B** The relative expression level of the *ITGB3* gene in mouse aortic tissue was detected. **C** The relative expression level of the *ITGB3* gene in the atherosclerotic cell model was detected. **D** The expression level of ITGB3 protein was detected by Western blotting. **E** The gray values of the Western blotting strips were statistically quantified. **F** *MiR-351-5p* and *ITGB3* gene 3'UTR targeted binding sequence and *ITGB3* gene 3'UTR mutation sequence. **G** Dual-luciferase activity report detection results. *P < 0.05, *ITGB3* WT + *miR-351* mimic group vs. any group; **H** Western blot was used to detect the protein silencing efficiency of ITGB3 after transfection of three siRNA groups.** I** Statistical quantification of H western blotting results. **P < 0.01, *P < 0.05, vs. control group. Animal and cell experiments, N = 3 per group

Figure 8A shows the results of the localization and detection of *miR-351* expression in cells in a FISH experiment. *MiR-351* was expressed in both the nucleus and cytoplasm, and the expression intensity of *miR-351* in the LDL + HG group was significantly increased. Figure 8B shows the expression level of the *ITGB3* gene in mice. Increases in the expression levels of the large *ITGB3* gene were observed in both the *miR-351*^*−/−*^ and H + *miR-351*^*−/−*^ groups, and the differences were statistically significant compared with the WT and HWT groups (P < 0.01). Figure 8C shows the expression level of the *ITGB3* gene in cells. The expression level of the *ITGB3* gene in the shRNA silencing group was significantly higher than that in the control group, and the difference between the two groups was statistically significant (P < 0.05). At the same time, the expression of the *ITGB3* gene was significantly decreased in the LDL + HG group (P < 0.05). The expression level of the *ITGB3* gene in the LDL + HG + shRNA group was substantially higher than that in the LDL + HG group (P < 0.05), and there was no significant difference between the control group and the LDL + HG + shRNA group (P > 0.05). The statistical quantification results of the western blots shown in Fig. 8D, E suggested that the protein expression level of *ITGB3* in *miR-351* knockout mice was significantly increased compared with those in the WT and HWT groups (P < 0.05). Therefore, we designed mutation sites of the *ITGB3*-3 'UTR for *miR-351* and the *ITGB3*-3' UTR (Fig. 8F). Combined with the dual-luciferase reporter assay, these results verified the targeting relationship between *miR-351* and *ITGB3*. The fluorescence intensity of the wild-type *ITGB3* WT group decreased significantly after transfection with the *miR-351* mimic (P < 0.05), but there was no significant change in fluorescence intensity in the mutated *ITGB3* MUT group. Therefore, *miR-351* may bind to the *ITGB3*-3 'UTR and degrade its mRNA. After siRNA targeting the *ITGB3* CDS region was designed, the cells were transfected. The inhibitory effect of siRNA was then detected by western blot (Fig. 8H, I). SiRNA3 had the most apparent inhibitory effect on ITGB3. In subsequent experiments, siRNA3 was used as the siRNA for *ITGB3* and denoted as siRNA for expression.

### Inhibition of ITGB3 expression can reverse the protective effect of miR-351 silencing on endothelial cells

**Fig. 9:**
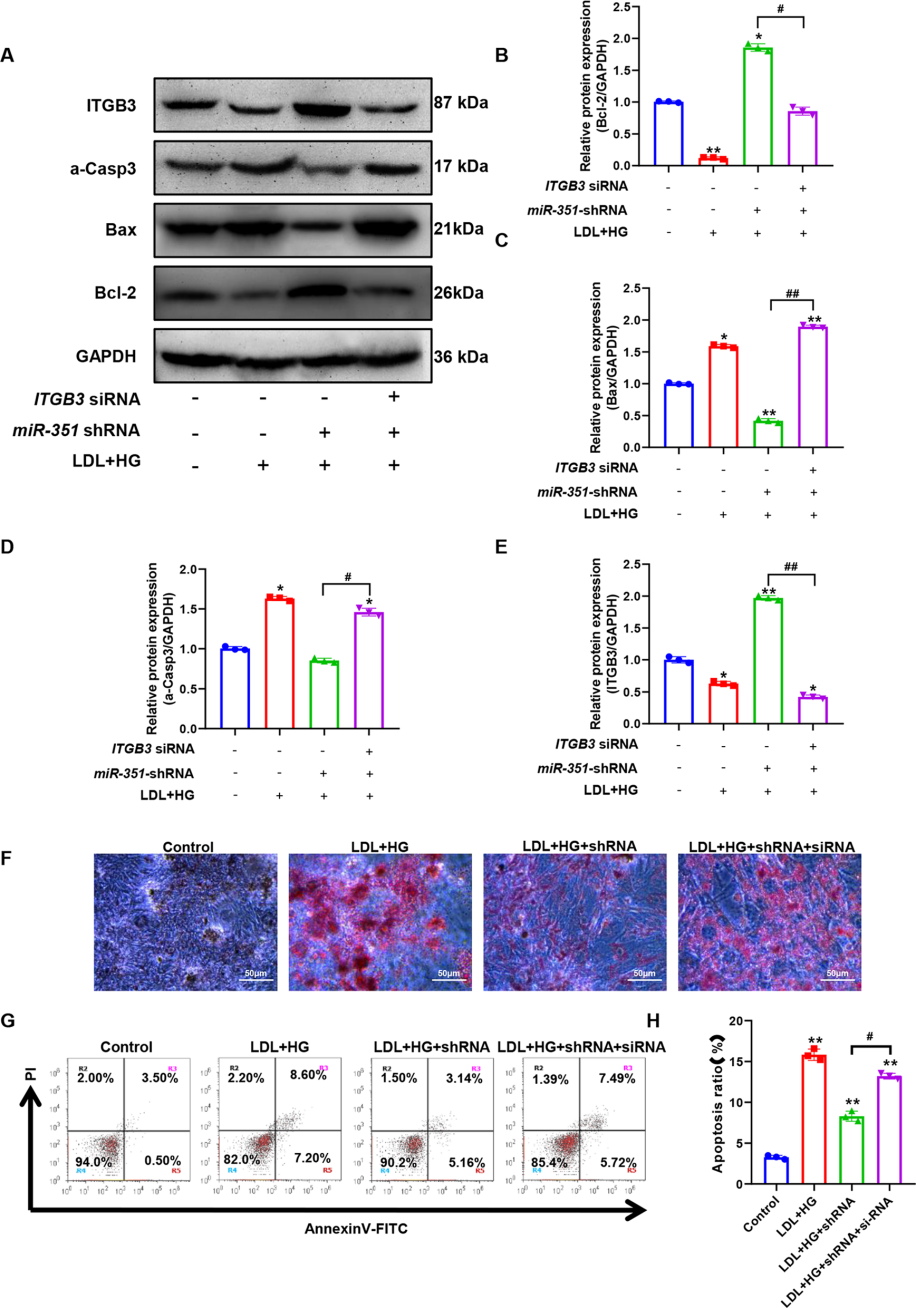
Effect of ITGB3 siRNA transfection on the biological function of *miR-351*-silenced cells. **A** The expression levels of ITGB3 and apoptosis-related proteins were detected by western blot. **B**–**E** The gray values of ITGB3 Bcl-2, Bax, and active Caspase-3 protein western blot bands were statistically quantified. **F** Oil red O was used to detect oil accumulation in cells. The scale bar represents 50 μm. **G** Flow cytometry was used to detect the apoptosis levels of *miR-351* cell lines after transfection with *ITGB3* siRNA.** H** The results were statistically quantified by flow cytometry. **P < 0.01, *P < 0.05, vs. the control group. ^#^P < 0.05 represents the comparison between the two groups of connections. N = 3 per group

Figure 9A shows the expression levels of apoptosis-related proteins and ITGB3 proteins detected by western blot. Figure 9B–E shows the results of statistical quantification of the gray values of Bcl-2, Bax, active Caspase-3, and ITGB3 protein immunoblot bands. Compared with the control group, the expression levels of the proapoptotic-related proteins Bax and active caspase-3 were significantly increased after LDL + HG induction. In contrast, the expression levels of the antiapoptotic proteins Bcl-2 and *ITGB3* were significantly decreased (P < 0.05). After silencing miR-351, compared with the LDL + HG group, the expression levels of the proapoptotic-related proteins Bax and active caspase-3 were significantly decreased, while the expression levels of the antiapoptotic proteins Bcl-2 and ITGB3 were significantly increased (P < 0.05). However, *ITGB3*-siRNA transfection effectively reversed the effect of *miR-351* silencing. Combined with the oil red O staining (Fig. 9F), *ITGB3*-siRNA transfection could change the reduction of lipid accumulation induced by silencing *miR-351* on LDL + HG. The results of apoptosis detection (Fig. 9G) were consistent with the results of western blotting detection, and the apoptotic cells decreased after silencing *miR-351*. The apoptosis level of *ITGB3*-siRNA transfected cells increased again, and there was no significant difference between *ITGB3*-siRNA transfected cells and the LDL + HG group (P > 0.05).

### Silencing miR-351 upregulates ITGB3 to activate Pik3r1/Akt pathway activity

**Fig. 10:**
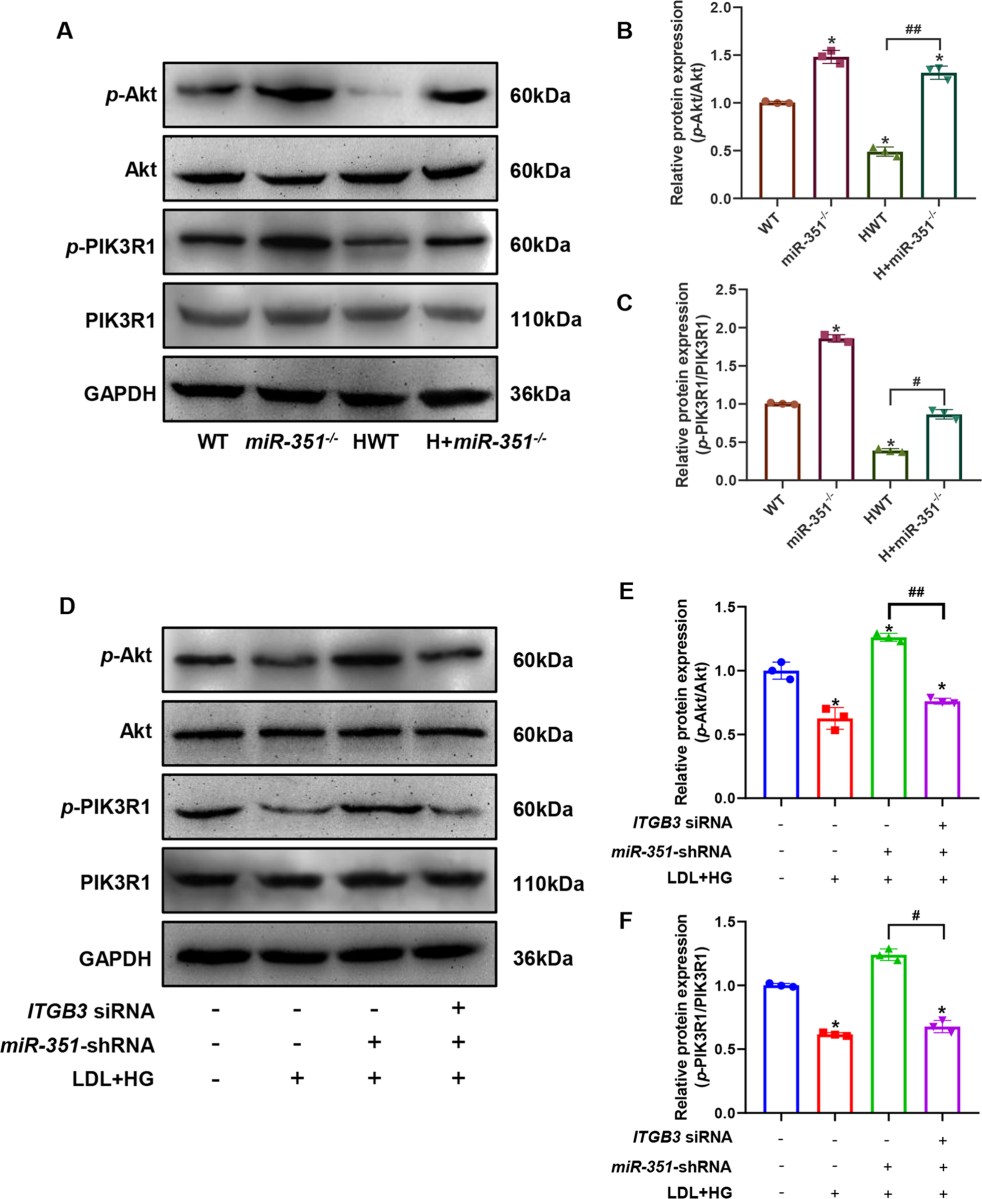
Protein expression level of the PIK3R1/Akt pathway detected by western blotting. **A** Results of western blot detection of mouse aortic tissue. **B**, **C** The western blotting bands quantified in mouse tissues are presented as the ratios of *p*-Akt/Akt and *p*-PIK3R1/PIK3R1. **D** Cell western blot detection results. **E**, **F** Quantitative statistical results of cell western blotting bands shown as the ratios of *p*-Akt/Akt and *p*-PIK3R1/PIK3R1. **P < 0.01, *P < 0.05, vs. WT group or control group. ^##^P < 0.01, ^#^P < 0.05, comparison between the two groups of connections. Animal and cell experiments, N = 3 per group

**Fig. 11:**
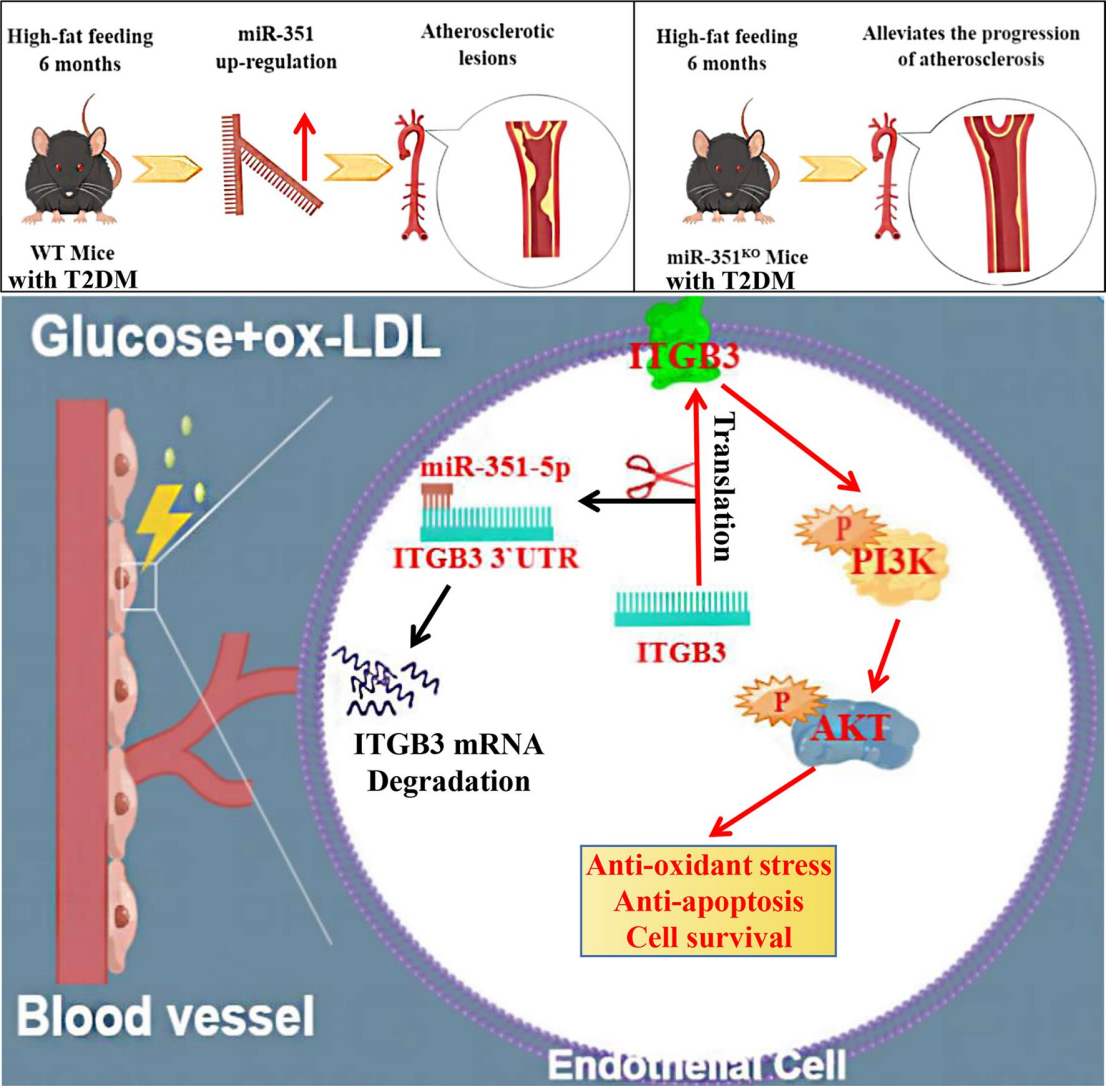
*miR-351* abolished *ITGB3* expression and the PI3K/Akt pathway in high-fat diet-induced atherosclerosis in T2DM mice. In the high-fat diet-induced atherosclerotic T2DM mouse model, *miR-351* was upregulated due to the influence of diabetes combined with a high-fat diet, while atherosclerosis was alleviated in the *miR-351* KO mouse model. The reason is related to the inhibition of *ITGB3* expression by *miR-351*. Downregulated *ITGB3* cannot stimulate the activation of the PI3K/Akt pathway, resulting in decreased cell resistance to oxidative stress and survival. After *miR-351* is eliminated, ITGB3's ability to stimulate the PI3K/Akt signaling pathway is restored, and endothelial cells can resist antioxidative stress and apoptosis and enhance cell survival

Figure 10A shows the results of the PIK3R1/Akt western blot in mouse aortic tissues, and Fig. 10B, C shows the quantified ratios of *p*-PIK3R1/PIK3R1 and *p*-Akt/Akt western blot bands in each group of mouse aortic tissues. The phosphorylation levels of Akt and PIK3R1 in the aortas of mice were significantly increased after *miR-351* knockout, and the phosphorylation levels of Akt and PIK3R1 in the HWT group were the lowest and were significantly different from those in the WT group (P < 0.05). Figure 10D shows the western blot detection of the PIK3R1/Akt pathway in the cell experiment. Figure 10E, F  shows the quantified ratios of *p*-PIK3R1/PIK3R1 and p-Akt/Akt western blot bands in cells with different treatments. The results showed significantly reduced ox-LDL- and high glucose-induced PIK3R/Akt pathway phosphorylation levels in endothelial cells compared with the control group (P < 0.05). The phosphorylation levels of Akt and PIK3R1 were significantly increased in *miR-351-*silenced cells and cells treated with ox-LDL and high glucose (P < 0.05). The *miR-351*-silenced cells were then transfected with *ITGB3* siRNA and induced with ox-LDL and high glucose. The results showed that *ITGB3* siRNA reversed the increased phosphorylation of Akt and Pik3r1 after silencing *miR-351*. The difference between the ox-LDL + shRNA group and the ox-LDL + shRNA + siRNA group was statistically significant (P < 0.05). Figure 11 shows the mechanism of action of miR-351 in diabetic atherosclerotic mice.

## Discussion

In this study, we investigated the role of *miR-351* in atherosclerosis. Both mouse and cell models of atherosclerosis showed high levels of *miR-351*. In addition, *miR-351* knockout mice were used to verify that *miR-351* knockout can slow the progression of atherosclerosis and lipid accumulation.

In the third month of atherosclerotic model induction, atherosclerosis was determined in 2 randomly selected HWT mice. Unfortunately, no evident atherosclerosis was observed in the aortas of these mice, and only minor damage to endothelial cells was observed. Therefore, we extended the study time of the whole experiment to 6 months. At the end of the investigation, more red spots were observed in the oil red O staining of the thoracic aorta of mice in the HWT group, but no large red plaques had formed. This result is similar to some earlier reports (Kunjathoor et al. 1996; Otero et al. 2005). Our study was also carried out in mice with diabetic atherosclerosis without using *ApoE* or *LDL* gene knockout mice, which can traditionally directly and significantly induce the formation of larger atherosclerotic plaques. We constructed mice with *mir-351* gene knockout, and to reduce the interference of more factors, no additional knockdown of the *ApoE* or *LDL* gene was carried out in mice, which led directly to the extension of the overall molding time.

From the later experimental detection, HWT group mice aortic endothelial cells and smooth muscle cells exhibited different degrees of abnormal proliferation, local vascular wall thickening, endothelial tissue protruding inward. Masson’s trichrome staining of vascular collagen showed less collagen in the HWT group. A study (Adiguzel et al. 2009) on collagen deposition in atherosclerotic vessels indicated that atherosclerotic sites are often associated with an aggregation of macrophages that secrete collagenase and gelatinase/type IV and V collagenase. Smooth muscle cells can also release collagenase and may participate in collagen degradation by releasing collagenase. We believe that this may be related to the accumulation of atherosclerotic macrophages. At the same time, our pathological results also showed abnormal proliferation of smooth layer cells. All of these factors could lead to collagen reduction in the HWT model group, similar to the study of (Wu et al. 2021) and (Jia et al. 2019).

In addition, silencing *miR-351* significantly reduced TG, TC, and LDL-c levels and increased the content of HDL-c in atherosclerotic mice. The expression levels of hs-CRP, TNF-α, NO and EDN1, *PTGIS,* and *AGT* genes at risk of atherosclerosis were significantly decreased. However, *NOS3*, an indicator of endothelial cell injury, was elevated, suggesting that *miR-351* silencing could provide endothelial cell protection (Förstermann and Münzel 2006). In addition, the experimental results showed that the indices (TC, TG, HDL-c, LDL-c, etc.) of KO mice and WT mice were similar before they were fed a high-fat diet, and there was no difference in the atherosclerotic phenotype due to miR-351 knockout. When endothelial cells are injured under conditions such as hyperlipidemia, it was found that miRNA could affect the process of atherosclerosis by regulating endothelial cell repair. However, in this study, a high-fat diet was not administrated in both wild-type mice and miR-351^−/−^mice, and no apparent endothelial damage was observed. Therefore, the phenotypic differences associated with atherosclerosis were not emphasized. These results indicate that silencing *miR-351* significantly reduces the degree of atherosclerosis. This is the first study to demonstrate that *miR-351* levels have been associated with atherosclerosis in most previous literature, without actual animal studies. We also verified *miR-351* expression in cell experiments. We used lentivirus infection to construct stable *miR-351*-expressing cells, which made the silencing efficiency of *miR-351* more stable, and we experimentally proved that *miR-351* silencing of mouse aortic endothelial cells can reverse the apoptosis induced by ox-LDL addition with high glucose. Similar to Zhang et al. (Zhang et al. 2015), the results reveled the significantly decreased survival rate after transfection of the *miR-351* mimic into endothelial cells of the cardiac aorta. However, their experiment still had some limitations, mainly because they did not induce an atherosclerosis model in cells and only explored the effect of *miR-351* on the survival of endothelial cells. The model of this study is an extension based on the atherosclerosis model, and the results are closer to the environment of the disease model. In addition, silencing *miR-351* reduced lipid accumulation and oxidative stress levels after silencing *miR-351*. In this study, the diabetic atherosclerosis model was induced by 30 mM glucose and ox-LDL. ROS levels were detected by flow cytometry, which proved that silencing *miR-351* could reduce ROS accumulation and oil accumulation and suppress oxidative stress levels.

Based on the above observation of *miR-351* in this study on the remission of diabetic atherosclerosis in mice and the protective effect of endothelial cells, we predicted the target of *miR-351*, which interacts with genes associated with endothelial cell protection, atherosclerosis, and diabetes. Integrin subunit β3 (*ITGB3*) was selected as the most relevant gene with a direct targeted binding effect. The *ITGB3* gene and protein levels were verified by the dual-luciferase activity reporting assay combined with the original *miR-351* knockout mice and *miR-351* silenced cell lines. These results demonstrated the targeted binding relationship between *miR-351* and *ITGB3*.

The ITGB3 protein product is the integrin β chain β3. Integrins are complete cell-surface proteins composed of α and β chains. A given chain may combine with multiple components to produce different integrins. Integrin β3 was found with αIIβ chains in platelets. Integrins are involved in cell adhesion and cell surface-mediated signal transduction (Alharbi et al. 2021). Overall, loss of *ITGB3*, which encodes integrin β3, exacerbates atherosclerosis in high-fat diet-fed *ApoE* (−/−) mice (Weng et al. 2003; Misra et al. 2018). In the context of atherosclerosis in *ApoE* (−/−) or *LDLR* (−/−) mice, *ITGB3* loss exacerbates HFD-induced atherosclerosis (Weng et al. 2003; Schneider et al. 2007). The femoral artery of *ITGB3-*deficient mice will collect more white blood cells after the arterial lumen is damaged, resulting in intimal hyperplasia (Smyth et al. 2001). All the above evidence suggests that *ITGB3* can slow the progression of atherosclerosis. Dahiya's study showed that apoptosis was increased after *ITGB3* mRNA and protein levels were downregulated by *miR-103b* (Danaei et al. 2011; Dahiya and Atreya 2021). This study also observed that the gene and protein expression levels of ITGB3 were significantly increased after the silencing of *miR-351*. Moreover, ITGB3 siRNA reversed the effect of silencing *miR-351* on cell apoptosis in atherosclerotic models. *ITGB3* siRNA can also change the effects of decreased lipid accumulation and proapoptotic proteins induced by *miR-351* silencing.

ITGA2B and ITGB3 proteins have been reported to be upregulated in serum exosomes in burn patients and are involved in injury detection and repair through the PIK3R1/Akt signaling pathway (Qin et al. 2020). ITGA2B and ITGB3 activate the PIK3R1/Akt signaling pathway and protect cells, especially nerves, as a cell survival signaling pathway (Xu et al. 2008; Yuan et al. 2011). The PIK3R1/Akt pathway is involved in cell proliferation, differentiation, and apoptosis (Qin et al. 2020). This highly activated pathway can strongly inhibit inflammation, reduce atherosclerosis, and promote angiogenesis. In atherosclerotic cell models, myricetin significantly attenuates ox-LDL-induced endothelial cell apoptosis by increasing the activation of the STAT3 and PIK3R1/Akt/NOS signaling pathways (Chen et al. 2014; Wang et al. 2011). Klotho improves oxidative stress induced by oxidized low-density lipoprotein (ox-LDL) by regulating PIK3R1/Akt/NOS pathways (Yao et al. 2017). Melatonin inhibits glucose-induced HUVEC apoptosis by activating the PIK3R1/Akt signaling pathway in a high-glucose-induced cell model. Akt activation further activates the Bcl-2 pathway by upregulating Mcl-1 and downregulating Bax expression to inhibit glucose-induced apoptosis of HUVECs (Tiong et al. xxxx). Therefore, we detected the phosphorylation level of the ITGB3/PIK3R1/Akt signaling pathway at the cellular level, and the results showed that the ITGB3/PIK3R1/Akt pathway in endothelial cells induced by ox-LDL plus high glucose was significantly inhibited, while the activity of the pathway increased after the silencing of *miR-351*. The transfection of *ITGB3* siRNA inhibited the phosphorylation of ITGB3/PIK3R1/Akt protein.

## Conclusion

In conclusion, we found that *miR-351* levels were significantly elevated in both mouse and cell models of atherosclerosis. Silencing *miR-351* can inhibit the progression of atherosclerosis and the occurrence of endothelial apoptosis events. In addition, we identified *ITGB3* as a direct target of *miR-351-5p.* In diabetic patients, miR-351 promotes atherosclerosis by targeting *ITGB3* and inhibits the PIK3R1/Akt pathway, upregulating endothelial cell apoptosis. These findings may provide potential therapeutic approaches for patients with diabetes mellitus with atherosclerosis. However, some shortcomings of this work need to be remedied by further research. This study only validated *ITGB3* as a target of *miR-351-5p,* but it remains to be studied whether *miR-351* can also regulate other targets involved in the regulation of atherosclerosis. Later detection should be added in patients with only atherosclerosis without diabetes to determine whether the *mir-*351/ITGB3/PIK3R1/Akt pathway is also affected.

## Supplementary Information

**Additional file 1. **qPCR primer design. Design of *mmu-mir-351*silent primers. Itgb3 siRNA primer sequence design. Design of dual luciferaseactivity reporting primers.

## Data Availability

The datasets used and/or analyzed during the current study are available from the corresponding author on reasonable request.

## Abbreviations

ox-LDL: Oxidized low-density lipoprotein
T2DM: Type 2 diabetes mellitus
CVD: Cardiovascular disease
