# Supplementary material for: miR-351 promotes atherosclerosis in diabetes by inhibiting the ITGB3/PIK3R1/Akt pathway and induces endothelial cell injury and lipid accumulation
Source: Mol Med. 2022 Sep 30;28:120. doi: 10.1186/s10020-022-00547-9 (PMC9523959; doi:10.1186/s10020-022-00547-9)
Supplement: Supplementary file 1 — Additional file 1. qPCR primer design. Design of mmu-mir-351silent primers. Itgb3 siRNA primer sequence design. Design of dual luciferaseactivity reporting primers. [file 10020_2022_547_MOESM1_ESM.docx]

1. **qPCR primer design：**

*Edn1* forward-5 '-CTA CTT CTG CCA CCT GGA CAT C-3', reverse-5 '-CGC ACT GAC ATC TAA CTG CCT G-3'.

*Ptgis* forward-5 '-GGA GAC AGG TCT CCT TGA GTT C-3', reverse-5 '-AAC ATC CGC TGA GTG GAC ACG A-3'.

*Agt* forward-5 '-AAC TCG CTC CTT CAG AAG CAG C-3', reverse-5 '-TTC CGC ACA GTC TCT GAA GGT G-3'.

*Nos3* forward-5 '-CGC AAG AGG AAG GAG TCT AGC A-3', reverse-5 '-TCG AGC AAA GGC ACA GAA GTG G-3'.

*Icam1* Forward 5 '-AAA CCA GAC CCT GGA ACT GCA C-3', reverse-5 '-GCC TGG CAT TTC AGA GTC TGC T-3'.

*Vcam1* forward-5 '-GCT ATG AGG ATG GAA GAC TCT GG-3', reverse-5 '-ACT TGT GCA GCC ACC TGA GAT C-3'.

*Itgb3* Froward: 5'-GTG AGT GCG ATG ACT TCT CCT G-3', Reverse: 5' -CAG GTG TCA GTG CGT GTA GTA C-3'. Reverse: 5'-ATG CCA GTG AGC TTC CCG TTC AG-3'.

*Gapdh* Froward: 5' -CAT CAC TGC CAC CCA GAA GAC TG-3', Reverse: 5'-ATG CCA GTG AGC TTC CCG TTC AG-3'.

*mmu-miR-351-5p* Froward 5 '-CCT GAG GAG CCC TTT GAG, Reverse 5' -GAA CAT GTC TGC GTA TCT C.

U6 Froward: 5 '-CTC GCT TCG GCA GCA CAT, Reverse Forward :5' -TTT GCG TGT CAT CCT TGC G-3 '

1. **Design of *mmu-mir-351* silent primers**

Oligo1: Forward-5'-CCG GTC CCT GAG GAG CCC TTT GAG CCT CGA GGC TCA AAG GGC TCC TCA GGG ATT TTT G-3', Reverse-5'-AAT TCA AAA ATC CCT GAG GAG CCC TTT GAG CCT CGA GGC TCA AAG GGC TCC TCA GGG A-3'.

Oligo2: Forward-5'-CCG GCC CTG AGG AGC CCT TTG AGC CCT CGA GGG CTC AAA GGG CTC CTC AGG GTT TTT G-3', Reverse-5'-AAT TCA AAA ACC CTG AGG AGC CCT TTG AGC CCT CGA GGG CTC AAA GGG CTC CTC AGG G-3'.

Oligo3: Forward-5'-CCG GCC TGA GGA GCC CTT TGA GCC TCT CGA GAG GCT CAA AGG GCT CCT CAG GTT TTT G-3', Reverse-5'-AAT TCA AAA ACC TGA GGA GCC CTT TGA GCC TCT CGA GAG GCT CAA AGG GCT CCT CAG G-3'.

Negative control：Forward-5'-CCG GCA ACA AGA TGA AGA GCA CCA ACT CGA GTT GGT GCT CTT CAT CTT GTT GTT TTT G, Reverse-5'-AAT TCA AAA ACA ACA AGA TGA AGA GCA CCA ACT CGA GTT GGT GCT CTT CAT CTT GTT G.

1. **Itgb3 siRNA primer sequence design**

siRNA1:SS-AAG AUC UUC GAA UCA UCU GGC. AS-CAG AUG AUU CGA AGA UCU UCU.

siRNA2:SS-ACA UGU ACG GCG AUA CAG GCU. AS-CCU GUA UCG CCG UAC AUG UAC.

siRNA3:SS-AUU GUA ACA GGG GUU UUU GAU. AS-CAA AAA CCC CUG UUA CAA UAU.

1. **Design of dual luciferase activity reporting primers**

Wild-type Itgb3 amplified primer sequence, ScaI-forward-5 '-AAA GAG CTC GCT TTC TCC TCA GGG GAG Agt AGT GC-3', XbaI-reverse-5' -TGC TCT AGA GTG TAG CAA GCA CAC AGA GGC A - 3 '.

Primer sequence of mutant Itgb3 amplification, ScaI-forward-5 '-AAA GAG CTC GCT TTG AGG AGA CCG GAG Agt AGT CC-3', XbaI-reverse-5' -TGC TCT AGA GTG TAG CAA GCA CAC AGA GGC A - 3 '. The product size was 125bp.
